# A High-Frequency Wearable IMU-Based System for Countermovement Jump Assessment

**DOI:** 10.3390/s26051408

**Published:** 2026-02-24

**Authors:** Antonio Pousibet-Garrido, Cristina Benavente, Juan A. Moreno-Pérez, Sergio Pérez-Regalado, Miguel A. Carvajal, Ignacio J. Chirosa, Pablo Escobedo

**Affiliations:** 1 PRIMELab, Department of Electronics and Computer Technology, Centre for Information and Communications Technologies (CITIC-UGR), University of Granada, 18071 Granada, Spain; antoniopoug@ugr.es (A.P.-G.); juanantoniomp@ugr.es (J.A.M.-P.); pabloescobedo@ugr.es (P.E.); 2 Sport and Health University Research Institute (iMUDS), University of Granada, 18016 Granada, Spain; cbenavente@ugr.es (C.B.); ichirosa@ugr.es (I.J.C.); 3 Department of Physical Education and Sport, Faculty of Sport Sciences, University of Granada, 18011 Granada, Spain; serperel@ugr.es

**Keywords:** countermovement jump, inertial measurement unit, wireless accelerometer wearable sensors, jump performance assessment, flight time, Internet of Things

## Abstract

**Highlights:**

**What are the main findings?**

- A fully custom wearable IMU-based system was developed for CMJ assessment using a single sensor sampled at 1 kHz.
- A derivative-based algorithm for take-off and landing detection improved the robustness of flight time estimation.
- The system showed high agreement with the force platform, with negligible bias and a detection rate above 97%.
- Data can be acquired and managed through both custom PC and smartphone applications and are automatically uploaded to a cloud platform.

**What is the implication of the main finding?**

- The proposed system provides a practical, accurate, and field-ready alternative to force platforms for monitoring CMJ in strength and conditioning and sport science environments.

**Abstract:**

The countermovement jump (CMJ) is widely used to monitor neuromuscular performance in sport, but its assessment is largely dependent on force platforms, which limits their use outside the laboratory due to their cost and limited portability. This work describes the development and validation of a fully custom wearable inertial measurement unit (IMU) system for CMJ assessment. The platform is based on a single IMU placed on the lower back and sampled at 1 kHz, and includes Bluetooth Low Energy (BLE) communication together with dedicated PC and smartphone applications. A new algorithm based on the derivative of vertical acceleration was implemented to identify take-off and landing instants. The system was evaluated using 119 CMJ trials performed by 19 participants and validated against a force platform used as the criterion reference. Different acceleration thresholds were tested, with 0.2 g providing the best compromise between detection robustness and the statistical quality of the measurements, yielding a detection rate of 97.43%. Agreement analysis showed a small systematic underestimation of flight time (bias = −0.0117 s), with moderate limits of agreement across the observed range. These results indicate that the proposed system may be suitable for practical, field-based CMJ monitoring, although the observed variability relative to force-platform measurements should be considered, particularly in applications requiring individual-level decision making.

## 1. Introduction

A fundamental aspect of athlete monitoring, strength and conditioning training, and applied sport science research in general is the evaluation of neuromuscular performance. The countermovement jump (CMJ) is one of the most popular performance tests used, mainly because of its simplicity, high test–retest reliability, and sensitivity to changes in lower-limb neuromuscular function. Previous studies have shown that CMJ-derived variables are very useful for monitoring fatigue, readiness, and training adaptations in various athletic populations [1,2].

A well-established criterion method for CMJ assessment is the use of force platforms, since they allow direct measurement of vertical ground reaction forces with high temporal resolution. These types of platforms allow one to derive jump height from impulse–momentum or take-off velocity approaches, which are considered biomechanically robust and less sensitive to technique-related assumptions [3,4]. Despite these advantages, the routine use of force platforms in real-life scenarios is often limited by their high cost, lack of portability, and spatial constraints. These limitations have caused a growing interest in alternative measurement technologies.

In this context, several video-based assessment tools have been proposed to analyze jump performance. For instance, Balsalobre-Fernández et al. [5] recorded CMJ performance using a digital camera (Casio Exilim FH-25, Casio, Tokyo, Japan) at a sampling frequency of 240 fps and determined flight time using Kinovea software. Flight time was obtained through frame-by-frame analysis, and a strong correlation was found with an optical measurement system used as the gold standard (OptoJump IR, Bolzano, Italy). More recently, Chiu et al. [6] estimated acceleration during the CMJ using Kinovea software and computer vision techniques to track body segments during the jump, also at a sampling rate of 240 fps. Lower sampling frequencies have also been tested. Reference [7] used a smartphone camera (Motorola Moto X4, Chicago, IL, USA) configured at 30 fps together with Kinovea software (v0.8.15) to determine flight time in vertical jumps, and compared the results with those obtained from a force platform (AMTI OR6-7, Watertown, MA, USA) operating at 200 Hz. The main limitation of video-based systems is that their temporal resolution is constrained by the camera sampling frequency. Consequently, achieving a temporal resolution of 1 ms or lower may require high-speed cameras, which can substantially increase costs.

To overcome such limitations and reduce costs, wearable inertial measurement units (IMUs) have emerged as a promising alternative for field-based CMJ assessment [8]. IMUs estimate jump performance using kinematic data derived from accelerometers and gyroscopes. Several studies have reported strong associations between IMU-derived and force platform-derived CMJ outcomes, particularly for jump height [9,10]. These findings suggest that IMUs may represent a viable alternative for monitoring jump performance outside of laboratory settings. However, as emphasized in previous methodological work, correlation coefficients alone do not provide sufficient evidence of measurement validity or interchangeability between systems [11].

More recent validation studies have shown that the agreement between IMU-based systems and force platforms is highly dependent on methodological and analytical factors, including sensor placement, sampling frequency, signal filtering, drift correction, and event-detection algorithms [8,12]. Several authors have reported the presence of systematic bias in IMU-derived CMJ variables, with the direction and magnitude of error varying according to the computational approach used. Importantly, such bias may not be evident when analyses rely exclusively on relative measures of association, reinforcing the need for absolute agreement analyses.

From an applied strength and conditioning perspective, the usefulness of any monitoring tool depends not only on its agreement with a criterion method but also on the magnitude and structure of its measurement error. Consequently, validation studies comparing IMU-derived CMJ outcomes with force platform measurements should extend beyond correlation-based analyses and include the evaluation of systematic bias, limits of agreement, and absolute error, as recommended in methodological literature [13]. Such analyses provide essential information regarding the measurement characteristics of a system and its potential applicability in real-world settings.

Therefore, the purpose of this study is to evaluate the validity of an inertial measurement unit and data analysis algorithms for the assessment of CMJ performance by comparison with a force platform as the criterion standard. Specifically, this study aims to quantify systematic bias, limits of agreement, and typical errors between methods under controlled conditions.

## 2. System Description

**Figure 1:**
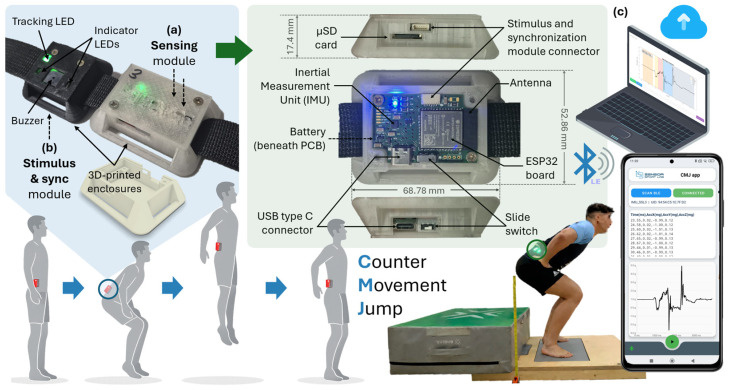
Overview of the developed IMU-based system for CMJ performance evaluation, composed of (**a**) sensing module; (**b**) stimulus and synchronization module; and (**c**) PC/smartphone applications with uploading to the MyoTrainer cloud platform.

The developed system is an IMU-based wearable platform designed for the assessment of CMJ performance in both field and laboratory conditions. A general overview of the platform is shown in Figure 1. The hardware part of the system is composed of two compact modules: a sensing module (Figure 1a) and a stimulus and synchronization module (Figure 1b). The sensing module mainly integrates the inertial measurement unit, the microcontroller and communication board (based on the ESP32), the battery, and a microSD (µSD) card for local storage of the acquired data. This sensing module is physically connected to the stimulus and synchronization module, which includes three LEDs (two indicator LEDs and one tracking LED, which is used for synchronization with the vision-based system adopted as the reference method), and a buzzer used to signal the subject to initiate the jump.

Both modules are housed in custom-designed 3D-printed enclosures with a buckle-type form factor, allowing them to be comfortably worn on the lower back using a standard belt. This configuration ensures stable positioning during the execution of the jumps while maintaining a high level of comfort for the athlete. As illustrated in Figure 1, the acquired data can be wirelessly transmitted via Bluetooth Low Energy (BLE) to a PC or a smartphone for data visualization, storage, and analysis. For this purpose, two custom applications have been developed: a desktop application and an Android smartphone application (Figure 1c).

In addition, the platform supports cloud connectivity through the MyoQuality+ cloud platform (https://myotrainerdevelopment.web.app, accessed on 1 February 2026, MyoQuality S.L., Granada, Spain), enabling remote data storage and access. This connectivity, together with the wearable sensing system and the companion mobile and desktop applications, defines an Internet of Things (IoT)-based architecture that enables wireless data acquisition and cloud-supported storage and analysis. Both hardware and software components of the system are described in detail in the following subsections.

The current platform represents a further evolution of our previously validated systems for CMJ and reaction time assessment [14,15], in which successive hardware and software iterations have been introduced. The present version builds upon the latest platform used for reaction time evaluation in athletics [14] and incorporates several additional improvements, including further miniaturization, enhanced wearability, the transition from Bluetooth Classic to BLE communication, an improved flight-phase detection algorithm, and overall usability enhancements for CMJ performance assessment when jump execution meets the criteria for mechanical efficiency [16]. Section 2.1, Section 2.2, Section 2.3, Section 2.4 and Section 2.5 describe the key hardware, firmware, and software improvements introduced with respect to the previous versions of the system. In addition, the proposed system is compared in the Section 4 with existing IMU-based CMJ solutions to emphasize the unique technical and methodological characteristics of the proposed system.

### 2.1. Hardware Architecture

The hardware architecture of the developed system has been redesigned to improve compactness, wearability, and usability while preserving the sensing capabilities required for accurate CMJ performance assessment. The sensing module consists of a custom-designed printed circuit board (PCB) with dimensions of 45 × 31 mm^2^, which integrates all the main electronic components in a single compact layout. This fully integrated design replaces the combination of commercial development boards and external communication modules used in earlier versions [14], resulting in a substantial reduction in size and weight. The compact PCB facilitates stable and comfortable placement of the device on the lower back, close to the body’s center of mass.

The sensing unit is based on the ICM20948 IMU (TDK InvenSense, San José, CA, USA), a 9-degree-of-freedom (DoF) sensor integrating a 3-axis accelerometer, gyroscope, and magnetometer. In comparison with the MPU-series sensors employed in the first CMJ prototype [14], this IMU provides improved performance and greater flexibility in terms of sampling configuration. For the purposes of the present study, only the accelerometer signals are used, allowing data acquisition at a high sampling frequency of up to 1 kHz for the three acceleration components, which is suitable for capturing the fast dynamics of the take-off and landing phases in CMJ tasks.

Data acquisition, processing, and communication are managed by an ESP32 microcontroller (ESP32-WROOM-32E-N16, Espressif Systems Co., Ltd., Shanghai, China). The ESP32 integrates a 32-bit processor and wireless communication capabilities within a single chip, enabling on-board data handling while maintaining a compact form factor. In the present design, communication with external devices is performed via BLE, replacing the Bluetooth Classic protocol used in the previous implementations [14,15]. This modification improves compatibility with modern mobile devices and contributes to reduced power consumption, which is particularly relevant for wearable applications. Power consumption was measured using a DC Power Analyzer (N6705A, Keysight Tech., Santa Clara, CA, USA) under the same conditions described in [15], at a constant supply voltage of 3.7 V. While the previous Bluetooth Classic implementation consumed 54 mA in idle mode, the current version maintains a comparable baseline consumption. However, in full-speed measurement mode (1 kHz streaming), current draw was reduced from 117 mA to 82.5 mA, corresponding to a 29.5% reduction. This improvement directly extends battery life during active measurement, thereby enhancing device autonomy for intensive monitoring scenarios.

The sensing module also includes a µSD card interface for local storage of raw acceleration data and processed results. This allows data logging independently of the wireless connection and provides a reliable backup during experimental sessions. Power is supplied by a 450 mAh lithium-polymer battery integrated within the enclosure. The charging and data interface has been updated from micro-USB to USB Type-C, improving mechanical durability, ease of use, and long-term reliability of the connector.

The sensing PCB is housed in a custom-designed 3D-printed enclosure with a buckle-type form factor, enabling direct attachment to a standard belt. The enclosure dimensions are 68.8 × 52.9 × 17.4 mm^3^, with a total weight of 37 g, which allows the device to be worn comfortably during CMJ execution without restricting movement. In addition, a dedicated external module was developed to host the stimulus and synchronization components. This stimulus and synchronization module (see Figure 1) integrates three LEDs and a buzzer in a compact and mechanically robust enclosure, with a total weight of 20 g. Compared with earlier implementations, the current design provides improved mechanical stability and more consistent positioning during use.

In summary, compared with the earlier CMJ prototype and the subsequent reaction time system, the present hardware architecture introduces several key improvements: a reduced PCB size, new lightweight enclosures optimized for belt attachment, the adoption of Bluetooth Low Energy communication instead of Bluetooth Classic, the replacement of the micro-USB connector with USB-C, and an overall reduction in power consumption. These modifications result in a more compact, ergonomic, and field-ready device, specifically adapted for routine CMJ performance assessment in applied sport science and strength and conditioning environments.

### 2.2. Firmware Description

The firmware has been completely redesigned to exploit the dual-core architecture of the ESP32 by assigning Core 0 exclusively to BLE communication tasks and Core 1 to IMU data acquisition. This parallel execution scheme allows the system to sustain a stable sampling frequency of 1 kHz while simultaneously transmitting data in real time, without blocking the acquisition process or relying on intermediate buffering in RAM or storage on the µSD card. As a result, the overall firmware architecture is simplified, and the microcontroller resources are used more efficiently. The main execution sequence is summarized as follows:

1. Core 0 initializes the BLE stack and configures the Maximum Transmission Unit (MTU) to 517 bytes. This MTU size allows the encapsulation of up to 6 data frames in a single BLE packet using a text-based format. Most devices supporting Bluetooth 4.2 or later are compatible with this configuration, ensuring broad interoperability with current smartphones and PCs.
2. Core 0 handles BLE write and notification events and manages the transmission of data packets. Data exchange between the two cores is performed through an inter-core queue, which provides a safe and efficient communication mechanism.
3. In parallel, Core 1 activates the tracking LED, initializes the IMU, and waits for the start command of the CMJ test.
4. Once the test begins, Core 1 starts sampling the 3 acceleration components at 1 kHz. An initial 0.5 s interval is recorded to establish a baseline of the acceleration signal before the buzzer is activated. After this, the buzzer is triggered to signal the subject to initiate the CMJ.
5. Acquired samples are grouped into data frames, and batches of six frames are pushed to the inter-core queue, from which they are immediately transmitted by Core 0 via BLE.
6. The data acquisition phase ends after a total duration of 3.8 s, after which the measurement is finalized.

**Figure 2:**
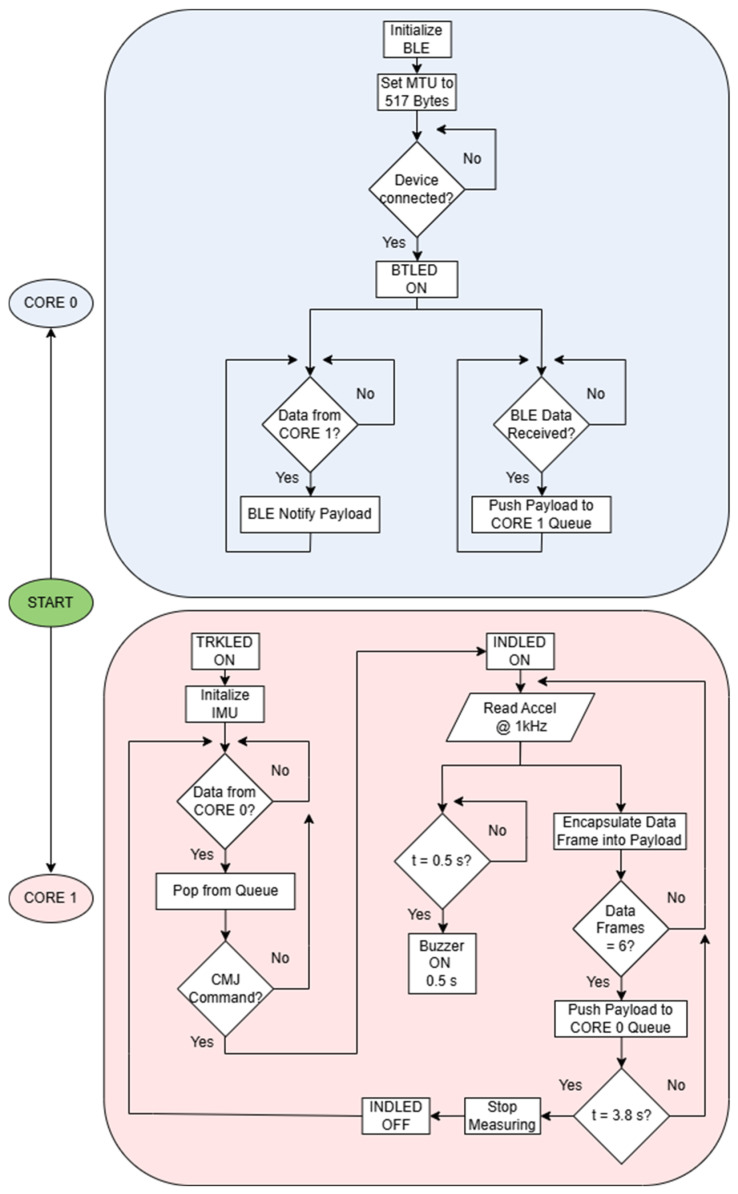
Firmware flowchart showing the parallel execution of IMU data acquisition and BLE communication tasks on the two cores of the ESP32. Abbreviations: BTLED: Bluetooth Status LED; INDLED: Indicator LED; TRKLED: Tracking LED.

This process is illustrated in the flowchart presented in Figure 2. It is important to highlight that the separation of acquisition and communication tasks across the two cores ensures that BLE transmission overhead never interferes with the 1 kHz sampling loop. Consequently, timestamps and acceleration data can be streamed reliably in text format without introducing timing jitter or data loss. In addition, the use of a text-based format simplifies data parsing on the client side and removes the need for dedicated decoding software, further improving the usability of the system in applied settings.

In comparison with the firmware described in our previous system [15], the current implementation makes use of the ESP32 dual-core architecture to decouple data acquisition from wireless communication. This redesign improves robustness during real-time streaming, reduces memory overhead, and ensures that BLE transmission does not affect the temporal integrity of the 1 kHz sampling process.

### 2.3. Algorithm for Flight Time and CMJ Phase Detection

In our earlier work on IMU-based jump assessment, a simple event detection algorithm was used, relying on local maxima and minima in the acceleration magnitude to identify take-off and landing instants [14]. However, due to the typical ringing and noise in inertial signals, this approach occasionally failed to detect jumps reliably, which will be discussed in the Section 4. In the present work, we propose and validate a novel algorithm that detects take-off and landing based on the derivative of the vertical acceleration, improving robustness and event detection accuracy over the previous method. The algorithm is based on the following main hypotheses:

- Hyp1: The most impulsive decrease in vertical acceleration occurs immediately prior to take-off (denoted t_DAccVmin_). At this instant, the absolute minimum of the derivative of vertical acceleration is expected.
- Hyp2: The most impulsive increase in vertical acceleration occurs immediately after landing (denoted t_DAccVmax_), producing an absolute maximum in the derivative.
- Hyp3: The flight time is determined as the interval of approximately zero vertical acceleration between t_DAccVmin_ and t_DAccVmax_.

**Figure 3:**
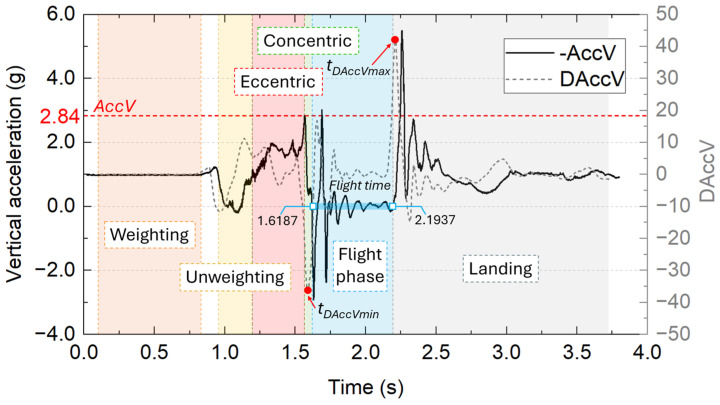
Example of one measured jump, representing the acceleration in the vertical direction (black solid line) and its derivative (gray dashed line). The different CMJ phases and main parameters are shown in the graph based on the proposed algorithm.

Due to the limited bandwidth and inherent ringing of IMU signals, establishing a strict zero-acceleration criterion is not always straightforward. Therefore, as described in detail later, several threshold values are evaluated to robustly define the flight interval in Hyp3 (an example of a measured jump is shown in Figure 3). Acceleration in the vertical direction (*AccV*) is sampled at frequency f_S_ = 1/T_S_, yielding a sequence of digital acceleration values as results of the jump monitoring. The derivate of the vertical component of acceleration (*DAccV*) is computed as the slope of a linear fit over a sliding window of samples:

(1) $$DAccV(n)=\left[\frac{dAccV}{dt}\right]={\left[Slope\{AccV,t\}\right]}_{n-N}^{n+N},$$

where *n* is the discrete time index expressed in multiples of the sampling time (*Ts*), and *N* the half-width of the sliding window, which has a size of 2N + 1 samples.

Using the developed system, the phases of the CMJ can be identified as follows:

1. Weighting phase: The subject is static, awaiting the auditory cue. During this phase the *AccV* should remain close to 1 g (baseline). From 0.5 s prior to the cue until the stimulus onset, the average (AVG) and standard deviation (SD) of *AccV* are computed. The weighting phase is considered to end when *AccV* exceeds the baseline by more than ten times the SD (*AccV* > AVG + 10 SD).
2. Unweighting phase: This phase begins when *AccV* crosses the baseline (1 g) with a negative slope, and it ends when *AccV* again crosses the baseline with a positive slope (See Figure 3).
3. Eccentric phase: It starts at the end of the unweighting phase and continues until the vertical acceleration reaches its local maximum (e.g., *AccV* = 2.84 g in Figure 3).
4. Concentric phase: The concentric phase extends from the local maximum of *AccV* until take-off.
5. Flight phase: The determination of the flight phase is conducted in two steps: first, the minimum and maximum in the *AccV* derivative (*t_DAccMin_* and *t_DAccMax_*) are identified within the jump interval; second, take-off and landing events are found as the times when *AccV* crosses a predefined threshold (*AccV_TH*) with negative and positive derivative, respectively. In cases where multiple crossings occur between *t_DAccMin_* and *t_DAccMax_*, the crossing closest to each derivative extremum is selected as the take-off or landing instant.
6. Post-landing monitoring: Following the landing event, acceleration is monitored until the acquisition period concludes (3.8 s in this work).

Compared with the algorithm used in our previous CMJ study [14], which detected take-off and landing from local maxima and minima of the acceleration magnitude, the present approach relies on the derivative of the vertical acceleration component. The previous method [14] was effective in most cases, but it was sensitive to signal ringing and noise, which could occasionally introduce spurious extrema and affect event detection. By focusing on the most impulsive changes in vertical acceleration, the derivative-based algorithm reduces this sensitivity and provides a more robust identification of take-off and landing instants.

One of the main design objectives of the proposed system was to maximize temporal resolution and effective bandwidth to minimize the ringing effects typically observed when low-pass filtering with low cutoff frequencies is applied. For this reason, accelerometer signals were not low-pass filtered prior to the computation of the acceleration derivative. Instead, the derivative of the vertical acceleration was estimated as the slope of a linear fit over a sliding window of 101 samples, which provides inherent smoothing while preserving impulsive features relevant for event detection. Acceleration was analyzed along the sensor axis corresponding to the nominal vertical direction (OY), based on a consistent sensor placement on the lower back. Gravity compensation was not applied, as this would require full orientation estimation using gyroscope and magnetometer data, resulting in a reduced sampling frequency. Accordingly, the signal processing strategy was designed to prioritize temporal precision and robust event detection.

### 2.4. Desktop Application

**Figure 4:**
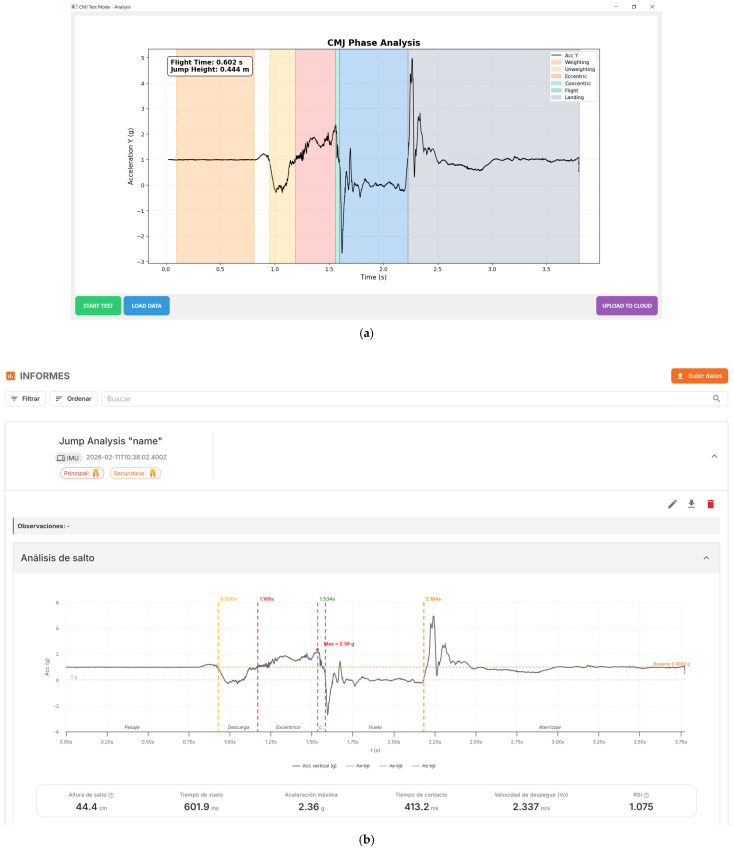
(**a**) Desktop application interface with CMJ phase analysis; (**b**) data uploaded to the MyoQuality+ cloud platform with the CMJ phase and the results of the jump.

A custom graphical user interface (GUI) was developed to manage the IMU device, visualize and log the acceleration data, and upload the results to the cloud. Communication with the sensing module is established via BLE version 4.2, which is supported by most modern personal computers. The developed application handles the BLE connection and provides a control panel to send custom commands to the IMU, display incoming data in real time, select the operation mode, and perform Over-The-Air (OTA) firmware updates. Figure 4a shows the CMJ phase analysis interface. When the user clicks on the START TEST button, the application sends a start command to the IMU, triggering the acquisition and transmission of acceleration data. The incoming data are temporarily buffered in the PC’s RAM and subsequently exported to a master spreadsheet template implemented in Microsoft Excel (Microsoft Corp., Redmond, WA, USA), which automatically computes the CMJ phases, flight time, and jump height [14]:

(2) $$H=\frac{1}{2}g\cdot {\left(\frac{t_{flight}}{2}\right)}^{2}$$

The vertical acceleration signal and the derived performance variables are simultaneously visualized in the embedded graph. In addition, the LOAD DATA button allows users to retrieve and inspect previously recorded tests, while the UPLOAD TO CLOUD button transfers the currently displayed data to the MyoQuality+ cloud platform for storage and further analysis, as can be shown in Figure 4b.

The desktop application was developed using Visual Studio Code (version 1.102.3) and Python 3.13 (64-bit). All tests were performed on an Asus TUF Gaming F15 laptop.

### 2.5. Smartphone Application

A dedicated smartphone application was developed to control the sensing module, receive and visualize the acceleration data. The mobile application is intended to complement the desktop software and to offer a practical and portable solution for field-based assessments, where the use of a PC or laptop may be less convenient. Communication between the smartphone and the device is established via BLE 4.2, which is supported by the vast majority of current smartphones. The use of BLE replaces the Bluetooth Classic communication employed in previous versions of the system, improving energy efficiency, connection stability, and compatibility with modern mobile operating systems.

As shown in Figure 1c, the application interface includes a scanning button to detect nearby devices and a visual indicator confirming successful connection. Once connected, the unique identifier of the IMU is displayed, allowing easy verification of the active device. A real-time data panel shows the incoming acceleration samples in text format, including timestamp and the three acceleration components. Below this panel, the vertical acceleration signal is plotted, enabling immediate visual inspection of the CMJ execution.

The central control button allows the user to start the acquisition process, triggering the test and the transmission of data from the IMU. This simplified interaction scheme minimizes operator intervention and facilitates fast repetition of trials during training or testing sessions. The combination of numerical data display and graphical visualization provides both detailed and intuitive feedback on signal quality and jump execution.

The smartphone application was developed using Android Studio Koala (version 2024.1.2) and is compatible with Android devices running Android 7.0 (API level 24) or higher. The application was compiled using Android SDK 34 and targets the latest Android platform version, ensuring compatibility with current smartphones.

## 3. Experimental Setup and Methods

### 3.1. Participants

A total of 19 male students from the Faculty of Sport Sciences at the University of Granada voluntarily participated in the study. The participants were aged between 18 and 25 (mean age: 21.5 ± 1.7 years) and presented the following anthropometric characteristics: height 1.78 ± 0.06 m, body mass 77.3 ± 6.8 kg and body mass index (BMI) 24.6 ± 2.8 kg·m^−2^. The participants had a consistent CMJ technique and were familiarized with the study protocol. To ensure a robust dataset, a total of 119 jumps were recorded across two sessions, separated by a minimum of 9 days. The physical variability among participants resulted in a wide range of flight times, from 0.469 s to 0.606 s, as measured by the force platform. This variability facilitated the validation of the developed IMU-based system across a representative range of jump durations.

Data collection was conducted at the Faculty of Sport Sciences, University of Granada (Granada, Spain) during May 2025. Each participant performed three valid CMJ trials per session. To achieve the total dataset of 119 jumps, two participants performed additional trials. Prior to testing, participants performed a self-selected warm-up to ensure readiness and minimize injury risk.

### 3.2. Experimental Setup and Protocol

The experimental setup comprised a force platform (serving as the criterion standard), the proposed IMU-based system, and a GoPro Hero 10 Black camera (GoPro, Inc., San Mateo, CA, USA) recording at 240 fps to visually validate the jump execution technique. The camera was mounted on a standard tripod and positioned perpendicular to the force platform axis. As illustrated in Figure 1, participants began in a static upright position on the force platform to allow for initial weighing and baseline signal acquisition. The inertial sensor was secured to the lower back (L5), corresponding to the lumbosacral junction, using a waist belt. The stimulus and synchronization module was attached to the right side of the belt, ensuring that the tracking LEDs remained within the camera’s field of view.

During testing, participants performed the CMJ technique by standing with the knees and hips fully extended, feet approximately shoulder-width apart and hands placed on the hips to minimize the contribution of arm swing. No explicit instruction regarding countermovement depth was provided, and participants were allowed to adopt a self-selected depth [17,18]. Participants were instructed to jump vertically and to land with both feet on the force platform, maintaining balance until the end of the recording period. Prior to data collection, participants were familiarized with the procedure through practice trials.

### 3.3. Data Acquisition and Processing

The measurement protocol proceeded as follows: Once the force platform successfully acquired the baseline signal, the IMU was activated to continuously stream triaxial accelerometry data via BLE at 1 kHz. After a 0.5 s delay, an auditory cue (4 kHz, 0.5 s duration) signaled the participant to initiate the jump. The IMU continued capturing data for a fixed window of 3.3 s, after which transmission was automatically terminated. Concurrently, the force platform recording was manually stopped by the operator once the movement was completed. The custom PC application then immediately processed the vertical acceleration (Y-axis) signal to calculate flight time, jump height, and jump phases. Finally, the results were displayed in real time, allowing the user to save the data locally or upload it to the cloud.

### 3.4. Data Analysis

Agreement between IMU-derived and force platform-derived flight times was evaluated using Bland–Altman analysis. Differences were computed as IMU minus force platform values. Systematic bias was defined as the mean of the differences, and the 95% limits of agreement were calculated as the bias ± 1.96 times the standard deviation of the differences. Confidence intervals for the bias and limits of agreement were computed assuming approximate normality of the differences, following the original Bland–Altman approach. The distribution of the differences was assessed visually using histograms and Bland–Altman plots.

## 4. Results and Discussion

The proposed IMU-based algorithm was quantitatively evaluated on 119 CMJ trials and benchmarked against the previous baseline approach. Several acceleration thresholds were tested (see *AccV_TH* in Section 2.3), and the force platform was used as the criterion (gold standard) method. The results of this comparison are summarized in Table 1.

The results in Table 1 illustrate the differences in performance between the previous algorithm and the proposed approach. While the previous algorithm shows a relatively low standard deviation (σ = 5.22%), its detection rate is slightly lower, with 8 valid jumps not being identified (93.16% success rate). The proposed algorithm improves this aspect, increasing the detection rate to approximately 98% across all tested thresholds.

**Figure 5:**
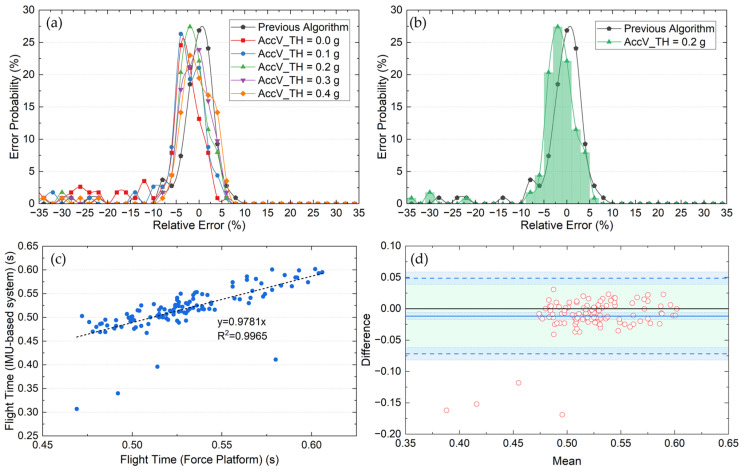
Performance evaluation of the proposed flight-time detection algorithm. (**a**) Relative error distributions for the previous algorithm and the proposed method using different acceleration thresholds; (**b**) Comparison of relative error distributions between the previous algorithm and the proposed approach using *AccV_TH* = 0.2 g; (**c**) Relationship between IMU-derived flight time (0.2 g threshold) and force platform measurements; (**d**) Bland–Altman plot assessing agreement between IMU-derived and force platform flight times using *AccV_TH* = 0.2 g.

The selection of this *AccV_TH* value is critical. Lower thresholds (0.0 g or 0.1 g) achieve the highest detection rate (98.29%) but introduce a considerable number of large errors greater than 10% compared with the gold standard system. Specifically, the 0.0 g threshold generates 23 large errors. This behavior is visually corroborated in Figure 5a, which shows an irregular distribution with secondary peaks in the tails. Consequently, these “bumps” degrades both the kurtosis (k = 2.24) and peak probability (24.57%), rendering this distribution unsuitable for practical implementation.

In contrast, the 0.2 g threshold (green line in Figure 5a) emerges as the optimal configuration. Despite a slight displacement of the mode toward the (−3, −1)% interval, it achieves the highest peak probability (27.43%) and kurtosis (k = 15.30). This represents a significantly sharper distribution than that of the previous algorithm (k = 11.18), indicating a higher concentration of measurements and reduced dispersion. Increasing the threshold further to 0.3 g or 0.4 g offers no operational advantage. These settings neither improve the detection rate (which remains constant at 97.43%) nor reduce the number of large errors (which remains at 4). Conversely, higher thresholds degrade the statistical quality of the distribution: the peak probability decreases from 27.43% at 0.2 g to 23.0% at 0.4 g, accompanied by a reduction in kurtosis.

A direct comparison between the previous algorithm and the optimal 0.2 g configuration is shown in Figure 5b. Although the proposed method introduces a slight negative bias, the histogram exhibits a more pronounced leptokurtic behavior. The 0.2 g distribution is taller and narrower than that of the previous algorithm (black line in Figure 5b), confirming that the new algorithm provides greater sensitivity and more improved repeatability under typical jump conditions.

The agreement between the IMU-derived flight times obtained with the 0.2 g threshold and those measured by the force platform was assessed using a Bland–Altman analysis (Figure 5d). The analysis revealed a small mean bias of −0.0117 s (95% CI: −0.0174 to −0.0060 s), indicating a slight systematic underestimation of flight time by the IMU-based algorithm. The 95% limits of agreement ranged from −0.0720 s to 0.0486 s, with corresponding confidence intervals of −0.0818 to −0.0621 s for the lower bound and 0.0388 to 0.0584 s for the upper bound, encompassing the vast majority of paired observations. Furthermore, no evidence of proportional bias was observed, as the differences were uniformly distributed across the range of mean flight times, suggesting that the level of agreement remained consistent for both shorter and longer jumps. Although four outliers with large positive deviations were identified, these isolated cases account for most of the observed dispersion. These outliers are the primary contributors to the standard deviation (σ = 5.98%) reported in Table 1. Visual inspection of the corresponding acceleration signals suggests that these large errors are most likely associated with transient sensor vibrations occurring during the flight phase, which interfere with landing event detection. Such signal perturbations are plausibly related to suboptimal IMU fixation on the lower back, a condition that may occur in realistic field settings. Aside from these cases, the remaining observations showed a stable level of agreement between methods, supporting the robustness of the proposed algorithm for CMJ flight-time estimation under the tested conditions.

To further examine whether the agreement between methods was consistent across individuals, an additional per-participant analysis was performed for the selected threshold (0.2 g). For each participant, the mean flight time error (IMU − force platform) was computed across their valid jumps. Per-participant mean errors ranged from −0.067 s to 0.006 s, with a median value of −0.005 s, which was consistent with the group-level bias. These results indicate that the overall agreement between methods was not driven by a small subset of participants, but was consistent across individuals. The largest negative value corresponded to a participant associated with two of the previously identified outliers.

For the selected threshold, a mean flight time of (0.518 ± 0.044) s was obtained from the 116 valid paired jumps recorded across the 19 participants, excluding three trials in which the force platform failed to trigger. The maximum and minimum values recorded by the IMU were 0.602 s and 0.307 s, respectively. Notably, the minimum value measured by the force platform was significantly higher (0.469 s). This discrepancy indicates that the minimum IMU value corresponds to a clear measurement artifact and is associated with one of the previously identified outliers. Overall, these results demonstrate that the dataset provided enough observations to validate the system across a representative range of flight times, reflecting the physical variability of the participants.

However, it should be noted that the present study was conducted in a relatively small sample of young, physically active male participants. As a result, the generalizability of the findings to other populations, such as female athletes, elite jumpers, youth, or older adults, remains to be established and should be addressed in future validation studies.

### Comparison with Existing IMU-Based Systems for CMJ Assessment

To contextualize the contributions of the proposed platform, a comparison with previously published IMU-based systems for CMJ assessment is presented in Table 2. This comparison highlights the main differences in terms of hardware configuration, sampling frequency, sensor placement, communication protocols, and user interaction capabilities. Such an analysis allows the distinctive characteristics of the present system to be discussed in relation to current state-of-the-art solutions.

Compared with previously reported IMU-based systems for CMJ assessment, the present platform integrates a distinctive combination of high sampling frequency (1 kHz), fully wearable standalone operation, BLE communication, and dual PC and smartphone interfaces. As shown in the table, the majority of existing systems operate at sampling frequencies between 50 and 100 Hz, with only a limited number reaching higher values. The proposed solution therefore provides a substantially higher temporal resolution while maintaining a compact and wearable configuration suitable for applied and field-based use.

An additional aspect that clearly emerges from the comparison is that most previous studies rely on commercial IMU platforms (e.g., Xsens, NAXSEN, Vicon, or Sensorize systems), which are primarily designed for general-purpose motion analysis rather than being specifically optimized for CMJ assessment. In contrast, only a smaller subset of works reports the development of fully custom systems. The present platform belongs to this latter category, as both the hardware and the software have been specifically designed and optimized for CMJ performance evaluation.

Although a limited number of recent studies have reported high sampling frequencies [26,32,33], these approaches are generally based on multi-sensor configurations or high-end commercial platforms primarily designed for laboratory biomechanical analyses. Such setups often involve increased system complexity, reduced portability, and higher costs. By comparison, the present system achieves a high sampling frequency of 1 kHz using a single IMU positioned at the lower back, preserving simplicity, portability, and suitability for routine CMJ monitoring in applied sport environments.

In summary, the comparison with existing IMU-based CMJ systems indicates that the proposed platform uniquely combines the following:

- A very high sampling frequency within a single-sensor wearable configuration.
- Sensor placement consistent with biomechanical best practices (lower back, close to the center of mass).
- A simplified signal model based exclusively on the vertical acceleration component.
- Modern and energy-efficient BLE communication.
- Dedicated user interfaces for both PC and smartphone operation.

The combination of these characteristics is uncommon in previous works, particularly among systems specifically developed rather than adapted from commercial IMU platforms, and underlines the originality and applied relevance of the proposed solution for CMJ performance assessment in strength and conditioning and sport science environments.

## 5. Conclusions

This work presents the design, development, and validation of a fully custom IMU-based wearable system for the evaluation of CMJ performance. The platform was conceived as an alternative to laboratory-based measurement systems, with a specific focus on achieving high temporal resolution, methodological robustness, and practical applicability in real-world environments.

The results show that a single IMU positioned on the lower back and sampled at 1 kHz, combined with the proposed derivative-based event detection algorithm, allows reliable identification of take-off and landing instants. The selected acceleration threshold (0.2 g) provided a suitable balance between detection robustness and statistical quality of the measurements, allowing estimation of flight time with reasonable agreement relative to the force-platform reference. Agreement analysis revealed a small systematic underestimation of flight time and moderate limits of agreement across the observed range of values. While this variability should be considered, particularly at the individual level, the proposed system represents a compact, practical, and field-oriented tool for field-based CMJ monitoring in sport science and strength and conditioning environments. Future work should evaluate performance in different populations and movement conditions to further establish generalizability.

## Figures and Tables

**Table 1:** Statistical performance and detection rate comparison between the previous system and the proposed algorithm under different acceleration thresholds.

Metric	Previous Algorithm	Acceleration Threshold (*AccV_TH*)				
		0.0 g	0.1 g	0.2 g	0.3 g	0.4 g
Mean (μ)	−0.88%	−6.49%	−3.32%	−2.22%	−1.74%	−1.29%
Median ($\tilde{\mu }$)	0.47%	−3.43%	−2.68%	−1.66%	−0.97%	−0.77%
Std. Deviation (σ)	5.22%	9.35%	6.32%	5.98%	5.99%	6.1%
Kurtosis	11.18	2.24	12.60	15.30	14.21	13.45
Mode interval	(−1, 1)%	(−5, −3)%	(−5, −3)%	(−3, −1)%	(−1, 1)%	(−3, −1)%
Peak probability	26.85%	24.57%	26.31%	27.43%	23.89%	23%
Correlation ($R^{2}$)	0.997	0.990	0.996	0.997	0.997	0.997
Error > 10%	4	23	7	4	4	4
Non-detected jumps	8	2	2	3	3	3
Valid jumps	108	114	114	113	113	113
Detection rate	93.16%	98.29%	98.29%	97.43%	97.43%	97.43%

**Table 2:** Comparison of IMU-based systems reported in the literature for CMJ assessment. N/A indicates that the corresponding information was not reported in the referenced study.

Type of System	Sensor Placement	F_s_ (Hz)	Signals Used	Comm.	User Interface	Ref.
Commercial (Sensorize)	Lower back (L5)	100	3D Acc + Gyro	BT	N/A	[9]
Custom system	Ankle	1000	Vert. Acc	BLE	Smartphone app	[19]
Commercial (Myotest SA)	Lower back	500	3D Acc	N/A	N/A	[10]
Custom system	Waist	300	3D Acc + Gyro	BT 2.1	PC application	[20]
Commercial (MTx, Xsens Tech.)	Lumbar spine (L4-L5)	100	Acc + Gyro + Mag	N/A	N/A	[12]
Commercial (VERT, Mayfonk)	Lower back (L3-L4)	N/A	3D Acc + Gyro	BT 4.0	iPad application	[21]
Commercial (MinimaxX S4, Catapult)	Back midline (T1-T5)	100	3D Acc + Gyro	N/A	PC application (Matlab)	[22]
Commercial (MicroStrain, Williston)	Back (L5)	100	3D Acc + Gyro	Wireless	PC application (Matlab)	[23]
Commercial (NGIMU, x-io Tech. Limited)	Back midline (L3-L5)	400	3D Acc + Gyro	N/A	N/A	[24]
Commercial (MTw Awinda, Xsens Tech.)	7 IMUs (lower extremities)	60	Acc + Gyro	N/A	PC (proprietary software)	[25]
Commercial (NAXSEN 2, SippLink Tech.)	Waist	1000	Vert. Acc	N/A	N/A	[26]
Commercial (FreePower, Sensorize)	Lower back (L5)	100	3D Acc + Gyro	BT	PC (proprietary software)	[27]
Custom system	Foot	200	3D Acc	Wi-Fi	PC application (LabView)	[28]
Custom system	Lower torso (T12)	50	3D Acc + Gyro	Wireless	PC application	[29]
Custom system	Lower back (L5)	300	3D Acc	Wi-Fi	PC application (Python)	[30]
Commercial (DOT, Xsens Tech.)	Lower back (L5)	60	Vert. Acc	N/A	PC application (Matlab)	[31]
Commercial (NAXSEN + Rabboni, SippLink)	Sacrum	1000 (USB); 50 (BT)	2D Acc	USB, BT	PC application	[32]
Commercial (Blue Trident, Vicon Motion Systems Ltd.)	7 IMUs (trunk, thighs, shanks, feet)	1600	3D Acc + Gyro	N/A	PC (proprietary software)	[33]
Custom system	Lower back (L5)	200	Vert. Acc	BT	PC/Smartphone applications	[14]
Custom system	Lower back (L5)	1000	Vert. Acc	BLE	PC/Smartphone applications	This work

## Data Availability

The data presented in this study are available on request from the corresponding author. The MyoQuality+ platform operates under the General Data Protection Regulation (EU Regulation 2016/679, GDPR). All users provide explicit informed consent prior to data collection. Athlete data are pseudonymized at the point of collection through unique identification codes.

## Abbreviations

The following abbreviations are used in this manuscript:

RT	Reaction Time
IMU	Inertial Measurement Unit
BT	Bluetooth
CMJ	Countermovement Jump
